# Functional syndromes and symptom-orientated aftercare after esophagectomy

**DOI:** 10.1007/s00423-021-02203-y

**Published:** 2021-05-25

**Authors:** Kristjan Ukegjini, Diana Vetter, Rebecca Fehr, Valerian Dirr, Christoph Gubler, Christian A. Gutschow

**Affiliations:** 1 Department of Visceral and Transplant Surgery, University Hospital Zurich, Zurich, Switzerland; 2 Department of General, Visceral, Endocrine and Transplant Surgery, Kantonsspital St. Gallen, St. Gallen, Switzerland; 3 Department of Endocrinology, Diabetology and Clinical Nutrition, University Hospital Zurich, Zurich, Switzerland; 4 Department of Gastroenterology and Hepatology, University Hospital Zurich, Zurich, Switzerland

**Keywords:** Esophagectomy, Functional syndromes, Functional aftercare, Quality of life, Dumping syndrome, Dysphagia, Delayed gastric emptying

## Abstract

**Background:**

Surgery is the cornerstone of esophageal cancer treatment but remains burdened with significant postoperative changes of gastrointestinal function and quality of life.

**Purpose:**

The aim of this narrative review is to assess and summarize the current knowledge on postoperative functional syndromes and quality of life after esophagectomy for cancer, and to provide orientation for the reader in the challenging field of functional aftercare.

**Conclusions:**

Post-esophagectomy syndromes include various conditions such as dysphagia, reflux, delayed gastric emptying, dumping syndrome, weight loss, and chronic diarrhea. Clinical pictures and individual expressions are highly variable and may be extremely distressing for those affected. Therefore, in addition to a mostly well-coordinated oncological follow-up, we strongly emphasize the need for regular monitoring of physical well-being and gastrointestinal function. The prerequisite for an effective functional aftercare covering the whole spectrum of postoperative syndromes is a comprehensive knowledge of the pathophysiological background. As functional conditions often require a complex diagnostic workup and long-term therapy, close interdisciplinary cooperation with radiologists, gastroenterologists, oncologists, and specialized nutritional counseling is imperative for successful management.

## Introduction

Esophageal cancer is the eighth most common malignant tumor worldwide, accounting for approximately 3% of newly diagnosed carcinomas [1–3]. In Western countries, the incidence of adenocarcinoma has significantly increased over the past decades, although the reasons for this shift are not fully understood [4, 5].

It is commonly accepted that surgical resection represents the critical component of oncologic therapy. In this context, the extent of surgery critically depends on both location and growth pattern of the tumor. Distal esophagectomy in terms of an extended gastrectomy is usually performed for Siewert II-III carcinoma of the esophago-gastric junction, whereas tumors located more proximally require subtotal or total esophagectomy with intrathoracic or cervical anastomosis. In most centers, standard reconstruction after esophagectomy is performed with a tubulized stomach, whereas a jejunal conduit as an esophageal substitute is an accepted alternative after distal esophagectomy. In contrast, reconstruction of the intestinal continuity with interposition of a colon segment is reserved for exceptional situations [6].

Perioperative morbidity and oncologic outcomes have long been the key measures of success in oncologic esophageal surgery. With the introduction of multimodal therapeutic strategies, long-term survival has significantly improved, and postoperative function and quality of life have increasingly come into focus in recent years [7]. Undisputedly, esophagectomy is a major and life-altering procedure, and less than 20% of patients report unimpaired postoperative alimentary comfort [8]. The most common functional conditions include dysphagia and reflux, dumping syndrome (DS), delayed gastric emptying (DGE), diarrhea, and weight loss. In view of a growing demand for competent functional aftercare, this review aims at providing a comprehensive summary of functional conditions after esophagectomy including an update on diagnosis and therapy.

## Dysphagia

### Pathophysiology and symptoms

Dysphagia is a common complaint after esophagectomy; however, only 3 to 4% of patients report clinically relevant symptoms [8, 9]. Stenoses or strictures of the anastomotic area are the typical underlying causes, but functional conditions—particularly after high cervical anastomosis—may also play an important role.

The pathogenesis of postoperative anastomotic stricture is not fully understood and probably multifactorial in terms of combined local ischemia and excessive anastomotic strain, promoting local inflammation, fibrin and collagen deposition, and consecutive scar formation [10, 11]. Therefore, dysphagia is considerably more common after anastomotic leakage [12–14].

**Fig. 1:**
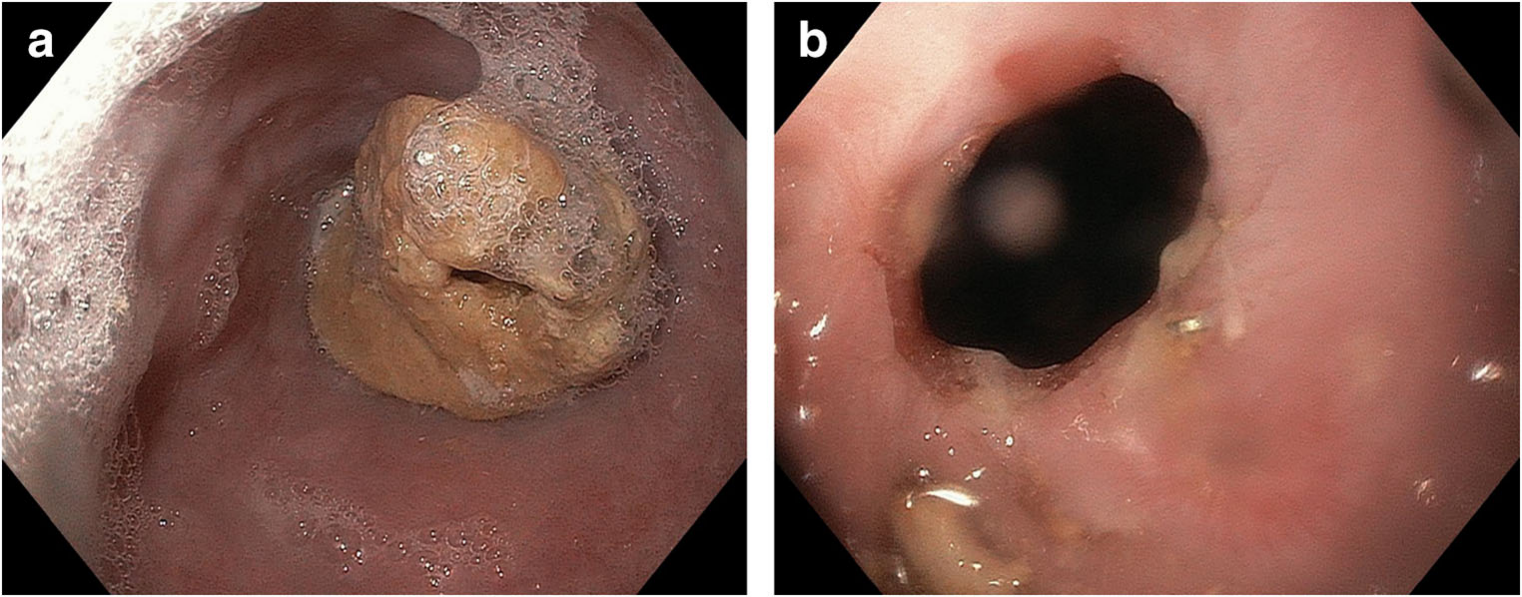
Anastomotic stricture after esophagectomy with gastric conduit reconstruction and intrathoracic anastomosis. **a**) Impacted food bolus **b**) Anastomotic stricture

On the other hand, new-onset dysphagia after longer follow-up is often caused by peptic strictures resulting from caustic reflux and insufficient acid suppression (Fig. 1), which in turn may be catalyzed by chronic DGE. Dysphagia occurs more frequently after cervical anastomosis than after intrathoracic reconstruction, although underlying issues are multiple and often functional. In this context, the anastomotic technique may have an impact on the incidence of dysphagia, as there is evidence of lower stricture rates after mechanical side-to-side anastomosis compared with circular stapler or manual suture anastomosis [15–17]. Likewise, a correlation between smaller circular stapler diameter (25 cm) and higher stricture rate has been evidenced in a systematic review [18]. Recurrent nerve palsy is also more common after cervical esophago-gastrostomy and confers an increased risk of aspiration [19].

Clinically, dysphagia may appear in varying degrees for all food consistencies, or exclusively for solids or liquids; the frequency of dysphagia also plays an important role. Therefore, detailed monitoring of symptoms is crucial. In this context, the Eckardt score—originally developed for the assessment of achalasia and its sequelae—allows for a straightforward and quick clinical evaluation (Table 1) [20].

**Table 1:** Eckardt score for clinical assessment of dysphagia (20)

Score	Symptoms			
	Weight loss	Dysphagia	Chest pain	Regurgitation
0	None	None	None	None
1	> 5 kg	Occasionally/weekly	Occasionally/weekly	Occasionally/weekly
2	5–10 kg	Daily	Daily	Daily
3	> 10 kg	Every meal	Every meal	Every meal

### Diagnostic workup and therapy

A targeted diagnostic workup for dysphagia after esophageal resection should start with endoscopy and contrast imaging. After exclusion of a mechanical cause or local recurrence, functional investigation of the laryngeal region including the upper esophageal sphincter with fiberoptic endoscopic evaluation (FEES) may provide further information.

Owing to their high restenosis rate, treatment of anastomotic strictures is a delicate undertaking. Endoscopic balloon dilatation or bougienage is currently considered the standard of care for benign anastomotic strictures [21, 22]. To avoid complications, strictures should be dilated by a maximum of three millimeter steps beginning from lowest resistance each time a dilation is performed [23]. Consequently, repeat endoscopies are often required for satisfactory results. Once an anastomotic width of more than 16 mm is achieved, patients can usually tolerate a normal diet [24]. In this context, simultaneous injection of corticosteroids may reduce complication rates and the number of dilatations required [25, 26]. In support, adequate acid suppression with proton pump inhibitors is strongly recommended. In the case of secondary caustic reflux as the cause of stenosis, the underlying DGE must be treated according to the measures discussed below in the section “Delayed gastric emptying.” Supportive dietary counseling including regular assessment of the nutritional status is crucial to prevent progressive weight loss. If oral caloric intake remains insufficient even after appropriate substitution with high-calorie sip feeds, insertion of a small-bowel feeding tube should be considered.

## Gastroesophageal reflux

### Pathophysiology and symptoms

Reflux symptoms after esophagectomy with gastric conduit reconstruction are reported by up to 60–80% of patients [27]. Often, symptoms manifest atypically in terms of coughing attacks, particularly when lying down or after meals. Several pathogenetic factors are discussed, including the loss of the natural antireflux barrier at the esophago-gastric junction and changed pressure conditions after transposition of the stomach into the negative thoracic pressure environment [27, 28]. In addition, the impaired motility and emptying ability of the gastric conduit in combination with a regenerating acid secretion over time may play an important pathophysiological role [29, 30]. Furthermore, after both intrathoracic [31] and cervical [32] reconstruction, the level of anastomosis appears to impact on both frequency and severity of reflux symptoms. In this context, low intrathoracic anastomosis should generally be avoided due to the higher risk of reflux problems [12]. As a consequence of chronic reflux, severe esophagitis and meta- and dysplasia in terms of a neo-Barrett’s esophagus may occur [33], and even de novo adenocarcinoma in the residual esophagus has been reported [34]. Nevertheless, the pathophysiological role of acidic and bilious reflux components has not been conclusively clarified [35].

### Diagnostic workup and therapy

Endoscopy remains the most important diagnostic modality, allowing for both macroscopic assessment and histologic clarification. Endoscopy also provides clues regarding underlying DGE, such as repeated detection of food residues in the gastric lumen after adequate fasting and a spastic pylorus. From our point of view, further diagnostic evaluation by means of esophageal and/or gastric long-term pH-metry or impedance monitoring is not routinely indicated owing to the lack of therapeutic consequences. However, a standardized assessment of symptoms is recommended; in this regard, the GERD-HRQL score according to Velanovich [36] (Table 2) and the health-related quality of life (HRQL) questionnaires published by the European Organization for Research and Treatment of Cancer (EORTC) [37, 38] have proven their clinical benefit.

**Table 2:** GERD-Health Related Quality of Life Questionnaire by Velanovich [36]

	Scale					
How bad is the heartburn?	0	1	2	3	4	5
Heartburn when lying down?	0	1	2	3	4	5
Heartburn when standing up?	0	1	2	3	4	5
Heartburn after meals?	0	1	2	3	4	5
Does heartburn change your diet?	0	1	2	3	4	5
Does heartburn wake you from sleep?	0	1	2	3	4	5
Do you have difficulty swallowing?	0	1	2	3	4	5
Do you have gassy or bloating feeling?	0	1	2	3	4	5
Do you have pain while swallowing?	0	1	2	3	4	5
If you take reflux medication, does this affect your daily life?	0	1	2	3	4	5
How satisfied are you with your current health condition?	Satisfied		Neutral		Dissatisfied	

Scale 0: no symptoms; scale 1: noticeable, but not bothersome; scale 2: noticeable, bothersome, but not every day; scale 3: bothersome daily; scale 4: bothersome and affects daily activities; scale 5: incapacitating to do daily activities

As a general recommendation after esophagectomy with gastric conduit reconstruction, the central therapeutic tool remains adequate long-term suppression of acid secretion with high-dose proton pump inhibitors (PPI). In severe or refractory symptoms, administration of H2-blockers or alginates may provide additional symptom relief. In suspected DGE as underlying cause of reflux symptoms, the specific endoscopic-interventional and conservative-prokinetic measures as described below should be followed.

## Delayed gastric emptying

### Pathophysiology and symptoms

Fifteen to 30% of patients after esophagectomy with gastric conduit reconstruction report typical symptoms of DGE [39]. The underlying mechanism is not fully understood, the most important pathophysiologic causes being impaired antro-pyloro-duodenal motility due to vagal and sympathetic denervation and transposition of the stomach to the thoracic negative-pressure compartment [40, 41]. In individual cases however, other factors such as diameter, volume, and redundancy of the gastric conduit, a prominent right diaphragmatic crus causing a kinked course of the conduit, the route chosen for reconstruction (posterior or anterior mediastinum), or a transhiatal prolapse of abdominal organs in terms of an enterothorax may be causative of DGE [42–44].

It remains controversial whether prophylactic intraoperative pyloric drainage via pyloroplasty or pyloromyotomy can lead to a significant reduction in the incidence of DGE. Moreover, with the introduction of minimally invasive surgical techniques, these procedures are used less frequently [45]. Reduced incidence of DGE after pyloric drainage has been evidenced in a meta-analysis [46], however without significant effect on the rate of other postoperative complications. In contrast, a more recent analysis of the literature [47] failed to prove significant effects of pyloric drainage, although the authors caveat that the lack of definition of DGE in the included studies significantly limits their power. Perioperative injection of botulinum toxin into the pylorus is a relatively new technique [48, 49]; however, further research is needed to prove its clinical value. The same applies to diversion of the tubular stomach by means of a Roux-en-Y or Billroth II jejunal loop [50].

The symptomatology of DGE ranges from early satiety, thoracic pain, and reflux symptoms to regurgitation and vomiting. However, the complex interplay of many potentially causative factors significantly complicates the clinical assessment and limits the evaluability of the current literature regarding incidence and the effectiveness of therapeutic interventions [42, 47]. In this context, the diagnostic criteria and the new grading system for DGE, published as part of an international consensus of experts, represent an important step forward (Table 3) [51].

**Table 3:** Scoring system for delayed gastric emptying by Konradsson et al. [45]

Questions:	Score			
Have you felt full up too quickly while having your meal during the past week?	0	1	2	3
Have you vomited during the past week?	0	1	2	3
Have you felt nausea during the past week?	0	1	2	3
Have you had acid, bile or food coming up into your throat or mouth during the past week.	0	1	2	3
Have you been unable to eat or drink enough to meet your daily need for energy during the past week?	0	1	2	3

Scale 0: not at all; scale 1: a little; scale 2: quite a bit; scale 3: very much

### Diagnostic workup and therapy

**Fig. 2:**
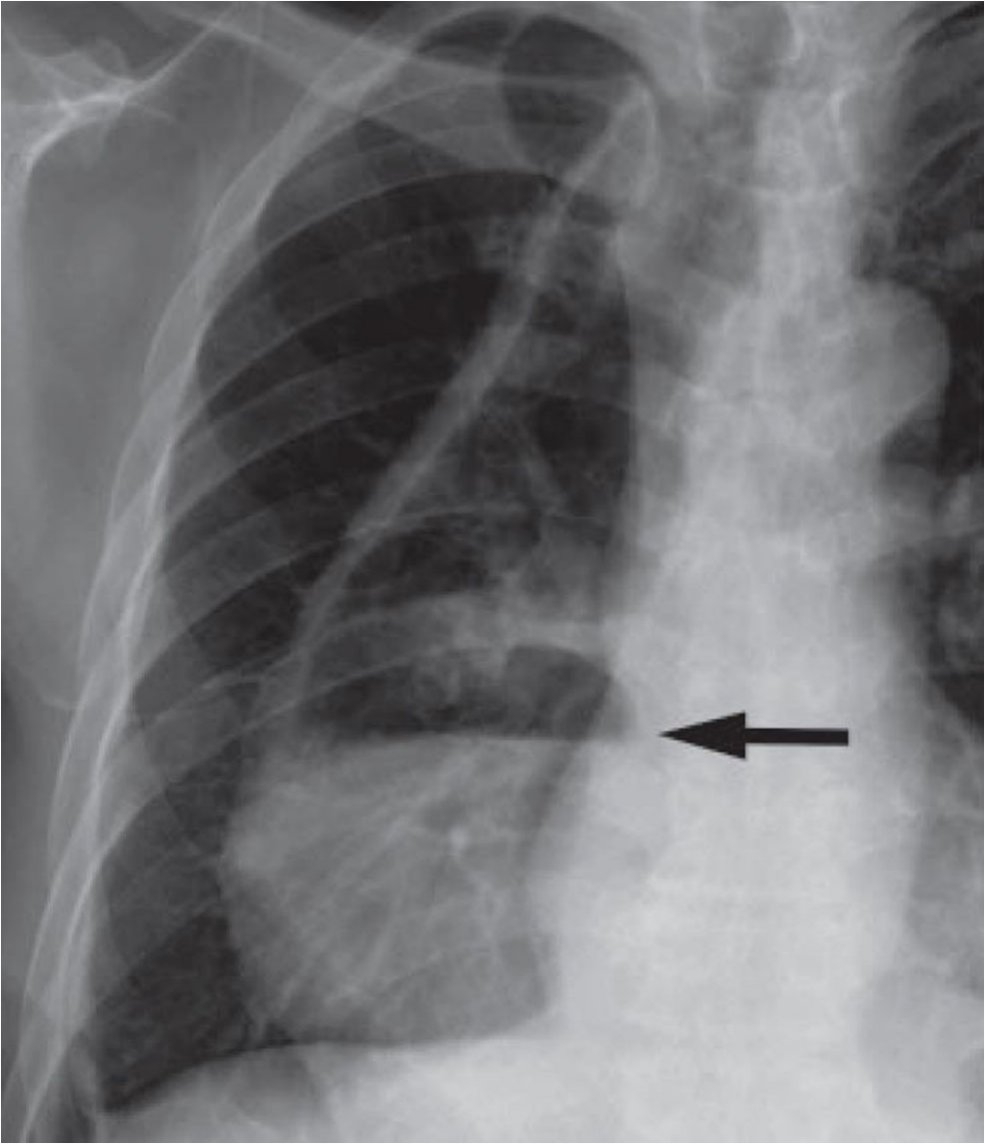
Plain chest X-ray showing a dilated gastric conduit with air-fluid level in a patient with DGE after esophagectomy and gastric conduit reconstruction

**Fig. 3:**
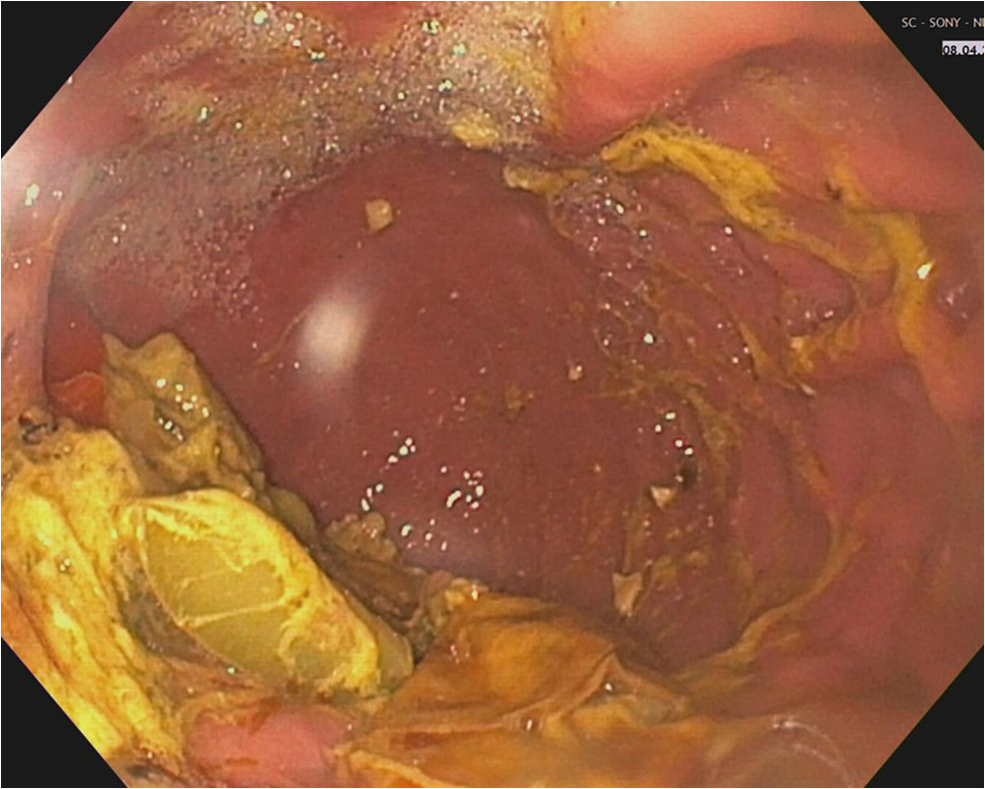
Endoscopic aspect of food residua in the gastric conduit in a patient with DGE

The first diagnostic step should focus on mechanical causes of DGE such as tumor recurrence or redundancy, kinking, or torsion of the conduit. Endoscopy and radiological investigations in terms of computed tomography and conventional contrast imaging are indicative in this regard. However, conventional chest X-ray often already reveals a characteristic pattern with a dilated conduit and air-fluid level (Fig. 2). During endoscopy, food residua in the gastric lumen after adequate fasting may be indicative for DGE (Fig. 3). In clinically unclear situations, a diagnostic attempt to detect gastroparesis can be made with gastric emptying scintigraphy [52] or a “smart pill” (Given Imaging, Yoqneam, Israel), which uses wireless transmission of pH data to indicate passage across the pylorus into the alkaline duodenal environment. Similarly, the ^13^C-octanoate breath test monitors the transpyloric passage of a ^13^C-isotope labeled meal. A new technique for planimetric assessment of pyloric distensibility has recently become available in the form of the Functional Luminal Imaging Probe (FLIP) [53, 54], although the clinical value of the test has not yet been conclusively established.

DGE can lead to relevant malnutrition, which is why competent nutritional counseling is generally recommended. As a rule, frequent small meals, a low-fat and low-fiber diet, and liquid or pureed foods are generally preferred since gastric emptying is often preserved for softer consistencies. In addition, a number of prokinetic medications are available for treatment of DGE: Metoclopramide is a widely used drug with propulsive and antiemetic properties. The propulsive activity is mediated by an antagonizing effect on the dopamine receptors of the enteric nervous system and increased release of acetylcholine from cholinergic neurons and sensitization of muscarinic receptors [55]. However, approval has been restricted in 2014 by the German Federal Institute for Drugs and Medical Devices because of potentially irreversible extrapyramidal parkinsonoid dyskinesia, which occurs in up to 10% of long-term users [56]. Domperidone has a similar mechanism of action, but less neurological side effects. However, dosage should not exceed 60 mg daily in elderly patients because of possible QT-time prolongation [57]. Erythromycin and azithromycin are macrolide antibiotics that are also used as prokinetic drugs due to their agonistic effect on motilin receptors, which are ubiquitously present in the GI tract. Erythromycin is considered a potent agent for accelerating gastric emptying and is therefore indicated in postoperative gastroparesis [58]. After esophagectomy and gastric conduit reconstruction, the combination of pyloric drainage with administration of erythromycin leads to a relevant reduction in bilious duodeno-gastric reflux [59]. Prucalopride is a relatively new drug, originally conceived for chronic constipation. It is a serotonin receptor agonist and chemically related to cisapride, but without its arrhythmogenic properties [57]. Prucalopride leads to an acceleration of gastric emptying as part of a general increase in gastrointestinal motility and can be used to treat DGE.

**Fig. 4:**
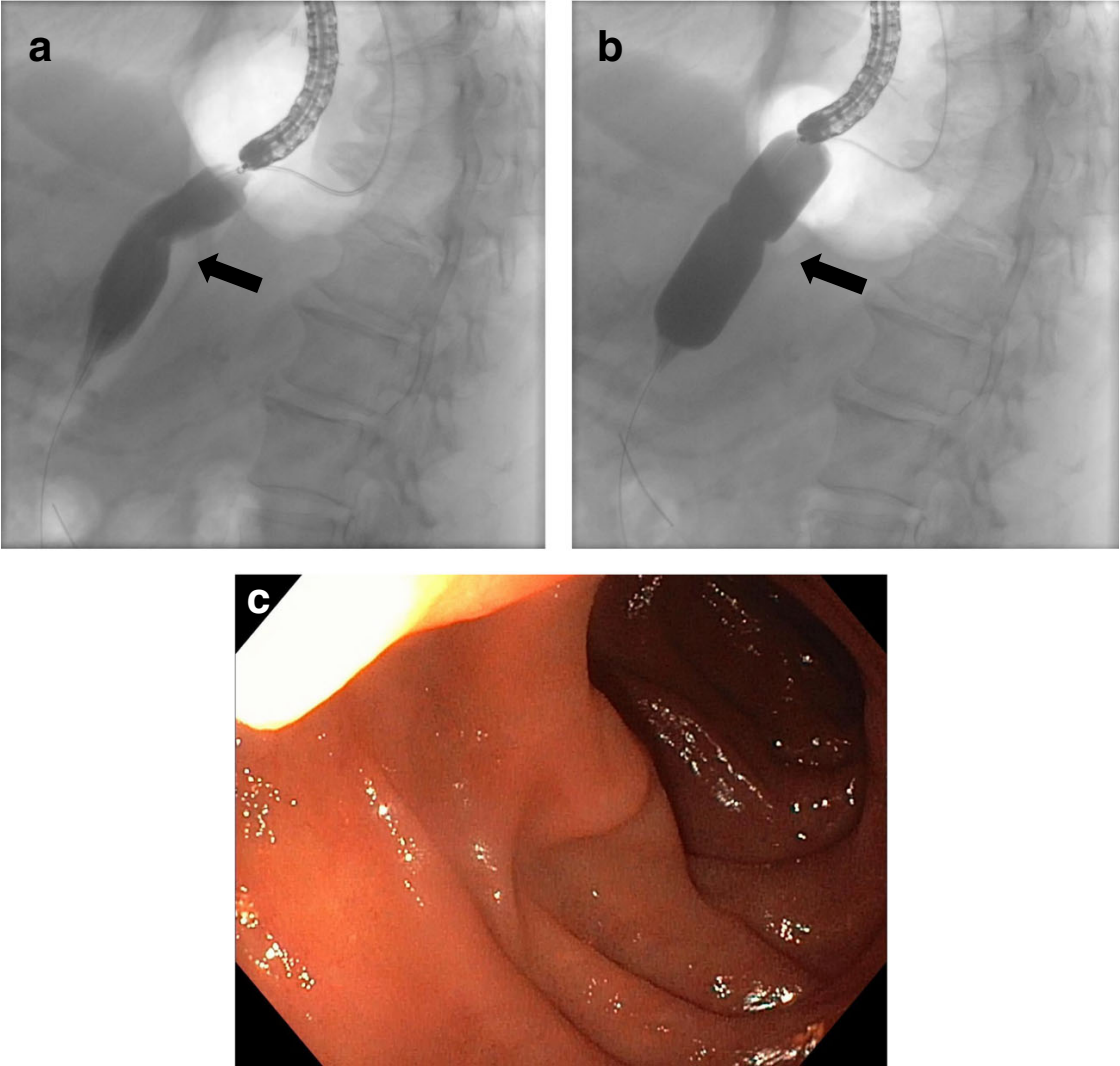
Fluoroscopy-guided endoscopic dilation of pyloric spasm causing DGE. The black arrow marks the pyloric region. **a** Before dilation, **b** balloon dilation, **c** endoscopic aspect after dilation

Interventional procedures to improve gastric emptying include dilation of the pylorus using balloons (Fig. 4) [60–63]; using the novel FLIP technique, dilation can also be performed in a controlled manner [64]. In contrast, perioperative injection of botulinum toxin into the pylorus aiming at improving postoperative drainage is not generally recommended. Other procedures for the treatment of DGE after esophagectomy include peroral endoscopic myotomy of the pyloric region (G-POEM) [65, 66] or the implantation of neurostimulators [67, 68]; however, the published evidence is too limited to make general recommendations [45]. In our own approach, diversion of the conduit with a jejunal loop according to Roux-en-Y has proven effective in individual situations [50].

## Dumping syndrome

### Pathophysiology and symptoms

DS is observed in up to 50% of patients after esophagectomy, with only 1–5% of patients showing pronounced symptoms [28]. The cause of DS is a lack of storage and accommodation capacity of the tubulized stomach plus vagotomy-related impaired antro-pyloro-duodenal motility. The above factors lead to unfractionated flooding of the small intestine with food and consecutive systemic reactions. In addition to the changes in anatomy and vagal innervation, disturbed neural and endocrine feedback via the osmotic and mechanical sensors in the small intestine also plays an important role. Thus, the interplay of a variety of gastrointestinal peptide hormones involved in digestion, such as GLP-1, CCK, PYY, PP, VIP, and neurotensin, is profoundly transformed after esophagectomy [69].

DS can clinically be distinguished into early and late dumping according to the timing of postprandial onset. Reported incidences of early and late DS vary from 40 to 70% and 20 to 40%, respectively, with frequent overlap between both syndromes [70].

Early DS (Table 4) is characterized by gastrointestinal and vasomotor symptoms, including bloating, abdominal pain, cramping, flatulence, diarrhea, circulatory problems, and even syncope. Symptoms usually start 10 to 30 min postprandially. Accelerated influx of hyperosmolar chyme to the small intestine results in a shift of fluid from the interstitial space to the intestinal lumen with consecutive increase of blood circulation in the splanchnic area, hypovolemia, hypotension, and a tendency to collapse. Mechanical stretching of the intestinal wall also results in vasomotor activation with compensatory activation of the renin-angiotensin system and hypersecretion of intestinal peptide hormones [71, 72].

**Table 4:** Signs and symptoms of early and late dumping syndrome

Early dumping symptoms		Late dumping symptoms
Gastrointestinal	Vasovagal	
Feeling bloated	Flushing	Sweating
Abdominal cramps	Headache	Flushing
Diarrhea	Loss of consciousness	Dizziness, lightheadedness
Nausea	Weakness	Rapid heart rate
Vomiting	Palpitations	Weakness
Borborygmi	Paleness	Tremor

In contrast, late DS typically occurs 1 to 3 h after meals with mixed symptoms of sweating, tremor, hunger, irritability, and lightheadedness. Due to the lack of pre-digestion, relatively large amounts of rapidly absorbable carbohydrates enter the small intestine, initially leading to hyperglycemia and followed by excessive insulin secretion with consecutive hypoglycemia [73–75]. Excessive release of GLP-1 may also play a role in reactive hypoglycemia; nevertheless, it remains ultimately unclear why only a proportion of patients becomes symptomatic [76].

### Diagnostic workup and therapy

DS is a clinical diagnosis made from the typical constellation of symptoms after gastric or esophageal surgery. Monitoring of the effectiveness of therapeutic measures can be performed with the Sigstad symptom score [77] (Table 5). In severe DS, it is recommended to perform an oral glucose tolerance test, in which symptoms, pulse rate, blood pressure, hematocrit, and serum glucose are monitored at 30-min intervals before and after glucose ingestion. Early or late DS can thus be detected with high sensitivity and specificity [78]. In contrast, scintigraphic gastric emptying or breath tests were not considered helpful in a recent expert consensus [79].

**Table 5:** Symptom score for dumping syndrome by Sigstad [77]

Symptoms	Sigstad score
Shock	+5
Fainting, syncope, unconsciousness	+4
Desire to lie or sit down	+4
Breathlessness, dyspnea	+3
Weakness, exhaustion	+3
Sleepiness, drowsiness, apathy, falling asleep	+3
Palpitation	+3
Restlessness	+2
Dizziness	+2
Headaches	+1
Feeling of warmth, sweating, pallor, clammy skin	+1
Nausea	+1
Abdominal fullness, meteorism	+1
Borborygmus	+1
Eructation	−1
Vomiting	−4
Total Sigstad Score	

Score >7: suspicion of dumping syndrome; score 4–7: no reliable assessment possible; score <7: no suspected dumping syndrome

The first therapeutic step should include nutritional counseling aiming at reduced carbohydrate intake and frequent small meals. In addition, liquids should be avoided during meals, as this accelerates food passage and increases the feeling of satiety. Complementary pharmacological approaches range from increasing chyme viscosity with guaran to the prescription of acarbose or diazoxide. Acarbose reduces rapid absorption of glucose by prevention of enzymatic cleaving of polysaccharides into smaller molecules. Diazoxide inhibits the secretion of insulin and is particularly indicated in late DS. Somatostatin or corresponding analogues may also be considered; however, the need for parenteral application is a major problem [28]. In this context, long-lasting somatostatin analogues with once monthly application have also shown to be effective [80].

## Nonspecific postoperative syndromes

Some of the most common symptoms and dysfunctions after esophagectomy cannot be clearly assigned and probably correspond to an overlap or a partial manifestation of the “classic” categories described above. In our experience, the most relevant nonspecific postoperative complaints are excessive weight loss with malnutrition on the one hand and persistent diarrhea or steatorrhoea on the other.

Even after an uncomplicated postoperative course and diet build-up, almost all patients experience unwanted weight loss after esophagectomy. The extent is typically related to the general nutritional status and is particularly pronounced in patients with severe preoperative weight loss, sarcopenia, advanced age, and vocal cord paralysis [81]. Consequently, early nutritional counseling is mandatory for preventing malnutrition during the initial postoperative phase. In our own practice, 5% weight loss over the first three postoperative months is tolerated without further intervention. Nevertheless, we strongly recommend careful screening of the nutritional status according to the ESPEN criteria (European Society for Clinical Nutrition and Metabolism) to monitor protein and caloric intake and to determine the need for nutritional supplements [82, 83]. In patients with a weight loss >5% or a BMI <18.5 kg/m^2^, close clinical monitoring and supplementation with high-caloric nutrition is indicated. If oral nutritional intake remains insufficient, we usually recommend insertion of a small-bowel feeding tube. Additional parenteral nutrition should only be performed in exceptional cases because of higher complication rates [84].

Chronic diarrhea and steatorrhea are frequent complaints after esophagectomy. Often, symptoms overlap with DS and require a similar diagnostic and therapeutic approach. Nevertheless, exclusion of infectious colitis or even a clostridial colonization by stool culture is generally indicated in this situation. In case of persistent chronic diarrhea limiting quality of life, symptomatic treatment with peristaltic inhibitors such as loperamide hydrochloride and probiotics should be considered. Similar to gastric resection, exocrine pancreatic insufficiency [85, 86] may lead to maldigestion and malabsorption with consecutive steatorrhea, meteorism, and intolerance of various foods. Determination of elastase-1 and stool fat content can provide additional information, and oral substitution of pancreatic enzymes is recommended.

## Health-related quality of life after esophagectomy

As with any malignant disease, the diagnosis of esophageal cancer has a significant impact on the overall and organ-specific health-related quality of life (HRQL). Curative therapy aims at curing the patient and restoring quality of life as completely as possible. However, due to the oncologic aggressiveness of the disease and the complexity of the surgical procedure—with significant changes to the upper gastrointestinal tract physiology—this goal is not always achievable [30, 87].

HRQL is defined as the extent to which physical, emotional, and social well-being is affected by a disease or its therapy [36]. Measurement of HRQL after surgical interventions has become increasingly important in recent years. Self-assessment scales, such as the EORTC quality of life questionnaires [88] and the Eypasch gastrointestinal quality of life index (GILQI) [89], are among the most frequently used.

There is general consensus that early postoperative quality of life is reduced after esophagectomy compared with preoperative levels [70] and healthy reference populations [90–94]. However, most authors agree that in the majority of patients, significant functional recovery takes place over the first 1–2 years [70, 95]. This dynamic can be explained both by a resolution of the immediate side effects of surgery during early recovery and by an increasing tolerance towards residual symptoms during long-term follow-up [38].

Nevertheless, the overall published evidence on mid- and long-term quality of life after esophagectomy is controversial, ranging from largely complete recovery without detectable deficits [9, 96–99] to permanent impairment [100–102]. However, the majority of studies show that most dimensions of HRQL remain reduced in the long term compared with healthy reference populations [94, 103–105], an effect apparently independent of surgical access routes or additional radio-chemotherapy or chemotherapy [37, 38, 94, 102, 103, 106, 107]. Similarly, there is no evidence for a significant influence of gender, tumor histology, or tumor stage on postoperative HRQL [37]. In contrast, postoperative complications such as anastomotic leakage may well have a sustained negative impact [94]. Nevertheless, most studies show that acceptable HRQL in the long-term follow-up after esophagectomy is possible in a high percentage of individuals [89, 90]. For example, in our own retrospective study, HRQL scores of 50% of patients >12 months after Ivor Lewis esophagectomy were at the same level compared with a healthy reference population. [38]

## Summary

**Fig. 5:**
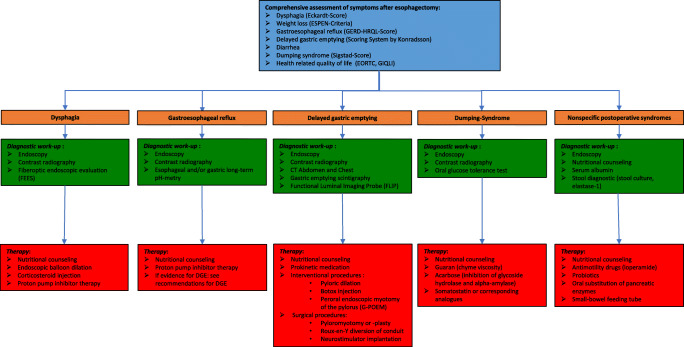
Diagnostic and therapeutic algorithm for functional syndromes after esophagectomy

Resection and replacement of the esophagus remain the critical components of curative therapy for esophageal cancer. Along with the progressive centralization of esophageal surgery in recent years, there has been a shift to highly specialized treatment in many Western countries. The increase in surgical expertise that came with this dynamic and the growing popularity of minimally invasive procedures has led to a reduction in perioperative morbidity and mortality [108] and probably also to an improvement in oncologic radicality [109]. However, despite the undeniable benefits of modern surgery, esophagectomy implies a considerable mutilation of the individual physiology, as the basic principles of surgical technique, which were established decades ago, remain largely unchanged [110]. Functional conditions after esophagectomy are highly diverse regarding clinical picture and individual expression and may be extremely distressing for those affected. Therefore, in addition to a mostly well-coordinated oncological follow-up, we strongly emphasize the need for regular monitoring of physical well-being and gastrointestinal function. The prerequisite for an effective “functional aftercare” that covers the whole spectrum of postoperative syndromes is the detailed and comprehensive knowledge of the pathophysiological background. As some functional conditions require a complex diagnostic workup and even long-term therapy, close interdisciplinary cooperation with radiologists, gastroenterologists, oncologists, and specialized nutritional counseling is imperative for a successful management (Fig. 5).
